# Indoor Location Technology for Managing Elective Surgery Patients in Hospitals

**DOI:** 10.1590/0100-6991e-20253813-en

**Published:** 2025-11-24

**Authors:** IBTISAM HAMZEH MOHAMMAD HUSEIN SHALABI, LAURA MARIA CÉSAR SCHIESARI

**Affiliations:** 1 - Fundação Getulio Vargas, Gestão em Saúde - São Paulo - SP - Brasil

**Keywords:** Health Management, Surgery Department, Hospital, Technology, Gestão em Saúde, Centro Cirúrgico Hospitalar, Tecnologia

## Abstract

**Introduction:**

With the increase in healthcare spending, efficient resource management in surgical hospitals has become essential, especially with regard to managing patient flow to avoid delays. Therefore, tools that can provide instant visibility of the patients location in each sector can be of great value in choices about resource allocation. Therefore, the objective of this study was to evaluate the applicability of indoor location technology in a hospital environment.

**Methods:**

Prospective study, carried out from February to March 2024, in a private surgical hospital, with tertiary structure, specialized in the care of elderly patients. The records of the path of each elective patient were mapped from their arrival to the moment of their discharge, as well as the activities of the professionals involved, through an indoor Bluetooth location device.

**Results:**

320 patients were analyzed, with an average stay time in the reception of 25 minutes and the time in the preoperative preparation unit of 107 minutes. Urology, mastology, and oncology surgeries represented 50% of the case series. The median transport time for these patients was 19 minutes. Reception (75.6%) and nursing admission (72.5%) were the sectors with the highest percentage of correct execution. The delay rate was 89.9% in the first time slot, and in subsequent times the delay was significantly lower (70.1%) compared to the time scheduled on the map.

**Conclusion:**

Indoor location technology has applicability when used in the intra-hospital environment in the management of surgical patients, facilitating the identification of bottlenecks and their causes

## RESUMO

**Objetivo:**

Com o aumento dos gastos em saúde, a gestão eficiente dos recursos em hospitais cirúrgicos se tornou fundamental, sobretudo no que diz respeito à gestão do fluxo do paciente para evitar atrasos. Portanto, ferramentas que consigam trazer visibilidade instantânea da localização do paciente em cada setor podem ser de grande valia nas escolhas sobre alocação de recursos. Por isso, o objetivo deste estudo foi avaliar a aplicabilidade da tecnologia de localização indoor em ambiente hospitalar.

**Métodos:**

Estudo prospectivo, realizado de fevereiro a março de 2024, em hospital cirúrgico privado, com estrutura terciária, especializado em atendimento de pacientes idosos. Os registros do trajeto de cada paciente eletivo foram mapeados desde sua chegada até o momento de sua alta, assim como as atividades dos profissionais envolvidos, através de dispositivo de localização indoor Bluetooth.

**Resultados:**

Foram analisados 320 pacientes, com tempo médio de permanência na recepção de 25minutos e o tempo na unidade de preparo pré-operatório de 107 minutos. Cirurgia de urologia, mastologia e oncológica representaram 50% da casuística. A mediana do tempo de transporte destes pacientes foi de 19 minutos. A recepção (75,6%) e a admissão de enfermagem (72,5%) foram os setores de maior percentual de execução correta. A taxa de atraso foi de 89,9% no primeiro horário, e nos horários subsequentes o atraso foi significativamente menor (70,1%) em relação ao horário programado em mapa.

**Conclusão:**

A tecnologia de localização indoor tem aplicabilidade quando utilizada no ambiente intra-hospitalar na gestão dos pacientes cirúrgicos, facilitando a identificação de gargalos e suas causas.

## INTRODUCTION

The Operating Room (OR) is one of the most complex hospital sectors, is widely exposed to possible complications, has low tolerance for errors and is responsible for a large part of the costs and revenues of hospitals. These factors create a scenario eager for monitoring solutions and constant search for efficiency. One of the great challenges is the definition of which efficiency indicators are the most important^1^ ^-^ ^3^. 

In the search for optimal efficiency in the OR domain, several authors describe that a fundamental strategy that enables the manager to better plan, organize, and coordinate this sphere is the adoption of Quality Indicators (QI) as a tool for evaluation and control in activities. QI should not be considered direct measures of quality, as they bring to light different factors that guide health care in a continuous process of quality improvement. Productivity indicators are more frequent in this context. Key factors in evaluating cost-effectiveness include the number of surgeries, cancellation rates and reasons, surgical schedule compliance, procedure size (duration), room turnover time, OR occupancy, average surgeries per room, surgery start-time average delay, and institution-specific metrics^2^ ^-^ ^6^.

Understanding the time spent at each stage of an elective surgical patient’s journey in the health unit can help understand the bottlenecks, both human and physical, and allow the visibility of unnoticed idleness in the management of a surgical hospital. An American study assessing 202 hospitals with the objective of understanding the resource consumption of patients undergoing elective abdominal surgeries through hospital cost centers found that bed occupancy was responsible for about 50% of all costs and is highly related to length of stay^7^. 

Currently, some hospitals and health centers have developed and applied technological systems for process improvement and bed management, which allow more efficient use of available resources^4^ ^-^ ^6^. An example is Location Based Service (LBS), driven by the popularity of smartphones, which people almost always have with them, opening many opportunities for using this tool in other areas such as medicine. The follow-up via LBS can improve interconnection within hospitals, manage personnel and equipment, analyze and optimize workflows, monitor the location of patients in emergency rooms and other overcrowded areas^6^ ^-^ ^8^. For these reasons, this study aimed to evaluate the applicability of indoor location technology in a hospital environment in the management of the elective surgical patient’s journey.

## METHODS

### Study Setting and Design

This is a prospective, quantitative study of an applied nature on the implementation of indoor technology in a hospital environment. We conducted it from February to March 2024, in a tertiary, private hospital in the city of São Paulo, state of São Paulo, Brazil.

The hospital is mostly surgical, with a verticalized operator, serving exclusively the adult public, attending surgeries of all anesthetic sizes on elective, urgent, and emergency bases. Its structure consists of an Operating Room with nine rooms, 12 day-hospital units, 95 inpatient beds, and 18 ICU beds. The study was approved by the Ethics in Research Committee (No. 6,621,500).

### Indoor Location System

**Figure 1A:**
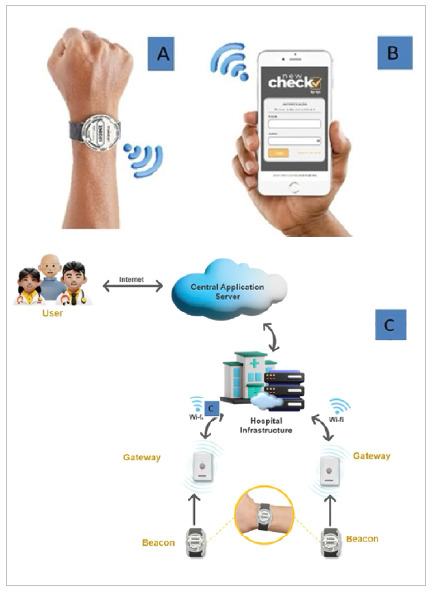
Beacon, BLE devices. 1B. Application used to associate the beacons with patients and record each stage of the surgical journey. 1C. Step-by-step of the Indoor location system.

We applied the indoor location system to each elective patient at the time of arrival at the hospital reception and the beginning of admission paperwork procedures. In this study, we used the combination of Wi-Fi and Bluetooth Low Energy (BLE), with the following hardware and software infrastructure (Figure 1):

1) Access Points make up the Wi-Fi infrastructure, distributing the signal throughout hospital environments.
2) Gateways, connected devices that pick up signals from Beacons (BLE).
3) Beacons, BLE devices, adapted in the form of watches and fixed to the patients’ wrists with a copy of the hospital identification bracelet, as illustrated in figure 1A.
4) New Check, an application used to associate beacons with patients, in addition to recording each stage of the surgical journey.
5) MQTT server, a messaging protocol specially designed for Internet of Things (IoT) devices.
6) MS-SQL Server, a relational database hosted on Microsoft Azure.

**Figure 2:**
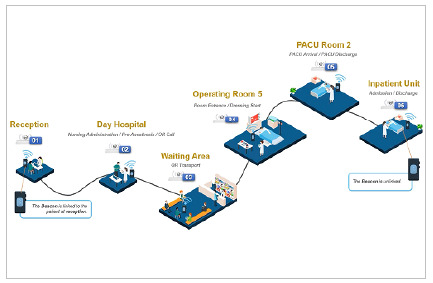
Detailed patient tracking in the hospital physical areas.

To evaluate and implement the necessary infrastructure, we mapped the patient’s path during the surgical journey, from entry at the reception to exit from the institution (Figure 2). The detailing of patient tracking in the physical areas of the hospital determined the number and distribution of gateways in the following hospital areas:

1) Reception;
2) Pre-surgery preparation area (Day Hospital or Long-Term Hospital);
3) OR area designated for preoperative patient preparation (preoperative PAR);
4) Operating rooms;
5) Post-anesthesia recovery (post-anesthetic PAR);
6) Hospitalization (Day Hospital or long-stay bed).

We evaluated and validated the hospital’s Wi-Fi coverage infrastructure throughout the journey. Gateways connected to Wi-Fi were installed, where positioning was used to capture and identify the beacons, determining patients’ paths. 

At every 20-second interval, the gateway picked up the signal from each detected beacon by measuring the received signal strength indicator (RSSI). After capture, RSSI data was temporarily stored in the device’s memory. Immediately after each scan cycle, this data was compiled and sent to the database via Wi-Fi. This process ensured continuous database updating with accurate information about time and location during the movement of beacons (patients).

The registration in the application allows the detailing of the activity carried out in a physical space, granting the understanding of when the patient is in a certain sector, if the main activity to be performed in loco has already been carried out, or is still pending.

**Figure 3A:**
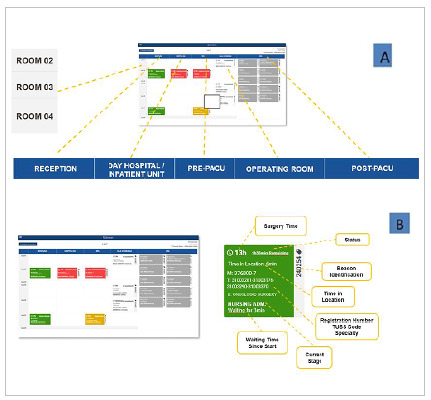
Real-time location of patients monitored on an online panel, with details. 3B. Detailing of the patient information card in real-time follow-up.

The patients’ path is visible to the sectors of interest through an online panel, allowing the entire care team involved to follow it (Figure 3).

### Eligibility criteria

#### Inclusion criteria

We included all patients electively scheduled in the surgical map who had the validation of the routes detected by the location technology and the data included in the application associated with the technology studied. As a criterion for considering the records in the app adequate, the study validated the position of the beacon associated with the patient at the time of record execution. 

We considered for the study all cases included in the research period in which the trajectory of the beacons was confirmed from their placement at the beginning of the elective surgical journey to discharge.

#### Exclusion criteria

FWe excluded all cases of non-elective patients (urgencies and emergencies), as well as individuals who had a new surgical approach, as it compromised the flow assessment in the sectors described. We also excluded cases in which there was a loss of signal, whether due to removal of the beacon or to failure in signal capture.

### Data collection

We measured and analyzed patients’ length of stay in each of the sectors of the hospital trajectory, from arrival to discharge. We assessed the activities recorded in the mobile application as to their execution in the correct place, for subsequent use of the data generated by the application (New Check).

We examined case demographics, surgical specialties, main procedures, and lengths of stay in both the reception and preoperative preparation areas across all specialties.

We analyzed the times of room occupancy and postoperative stay in the hospital after leaving post-anesthetic recovery for the specialties globally and through subgroups of procedures of these specialties, due to the great difference in procedures behavior even within specialties. 

Subgroups were defined within specialties where more than two cases could be grouped based on these procedures, allowing analyses to be conducted according to the complexity specific to each specialty, such as urology or oncological surgery.

Continuing with the analysis of the surgical journey, we divided the procedures into first-time surgeries and sequence surgeries, where we sought to study the rates of delay in the first time and correlations with the arrival times in the pre-procedure RPA sector more than 10 minutes before the scheduled time on the map.

### Statistical analysis

We present descriptive data by absolute (n) and relative (%) frequencies for categorical variables, and by median and interquartile range (IQR) for continuous ones. Upon application of the Shapiro-Wilk and Komogorov-Smirnov normality tests, the data showed a non-normal distribution, so we performed nonparametric tests for the analyses. We used the chi-square test to compare the frequency between variables. The analyses were performed using the SPSS software, version 21, considering a significance level of 5% (p<0.05).

## RESULTS

### Series

In the period between February 5, 2024 and March 5, 2024, 630 patients were scheduled for elective surgeries. Of these, 320 patients were selected to compose the final sample, since their paths were considered complete and were then validated. The general characteristic of patients was the mean age of 69 years (IQR 21-97), with 60.4% females and 39.3% males.

### Indoor System Data

The median length of stay in the admission stage was 25 minutes (IQR 14-38) and the median time in the preoperative preparation unit was 107 minutes (IQR 71-157). 

Of the 320 patients, 116 had the OR call app execution completed correctly (the tool must behave as an agile and effective communication instrument, activating the transport team immediately after filling out the application). The median transport time of these patients, appropriately signaled by the analyzed instrument (New Check app), from the preoperative preparation sector to the operating room, was 19 minutes (IQR 11-31) (Table 1). 

**Table 1:** Pre-surgical hospital times.

General pre-surgical times	n	Median	IQR
Admission	297*	25 min	14-38
Pre-surgical preparation	320	107 min	71-157
Transport call	116	19 min	11-31

IQR=Interquartile range. *The number of patients registered at reception was lower because 23 of them had their beacons activated in the preoperative preparation unit, with the entire subsequent trajectory complete, and were then included in the study.

**Figure 4:**
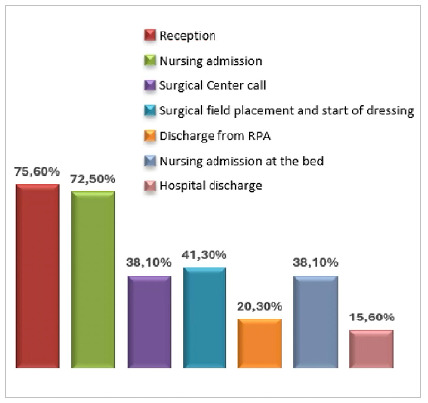
Percentage of use of the application at the time concomitant with patient care according to indoor location.

The analysis of the correct documentation of the steps in the application by the employees of each sector, referring to the activities performed, varied depending on the performing sector. Reception (75.6%) and nursing admission (72.5%) were the sectors with the highest percentage of correct execution. Figure 4 shows the other percentages of execution compliance.

**Figure 5:**
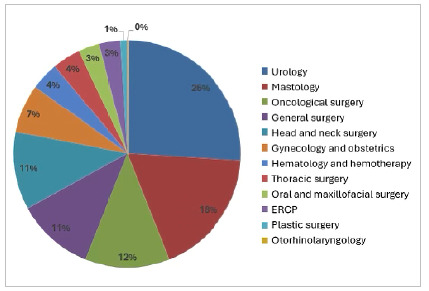
Description of the medical specialties analyzed. ERCP: endoscopic retrograde cholangiopancreatography..

The analysis of the specialties indicated that most of the cases included were from Urology, Mastology, and Oncological Surgery, together representing more than 50% of all cases. The other specialties are represented in Figure 5.

Room occupancy times represent the physical presence of the patient in the environment in question and are presented through the overall time of the specialty and its surgical procedures with greater volumetry, with quantification in minutes. In Urology, the cases with the highest volume refer to laparoscopic radical prostatectomies (n=20), with a median room time of 201 minutes, and endoscopic resection of bladder tumors (n=15), with a median of 83 minutes. In Mastology, the highlights are cases of quadrantectomies, with or without lymphadenectomy (n=35), with a median occupancy of the operating room of 123 minutes. The details of the other specialties are described in Table 2.

**Table 2:** Surgical specialties and their procedures.

Surgical specialties and procedures		n	OR time (minutes)	
			Median	IQR
Urology		83	138	87-199
Laparoscopic radical prostatectomy		20	201	190-239
Bladder tumor - endoscopic resection		15	83	64-91
Prostate electrovaporization		9	132	106-179
Percutaneous nephrolithotripsy		9	188	169-210
Nephrectomy		6	227	188-255
Unilateral flexible ureterorrenolithotripsy		6	97	74-135
Cystoscopy and/or urethroscopy		5	50	44-97
Endoscopic resection of the prostate		4	107	73-266
Radical cystectomy		2	291	250-291
Other		7	88	77-146
Mastology		58	125	99-156
Quadrantectomies with or without lymphadenectomy		35	123	97-139
Radical mastectomy		8	138	116-197
Mastectomy or quadrantectomy with immediate reconstruction		7	192	131-276
Excision of breast lesion		5	108	74-154
Other		3	115	77-115
Oncological surgery		39	160	120-263
Colectomy and rectosigmoidectomy		12	173	132-258
Oncogynecological surgery		10	135	115-161
Hepato-pancreatic surgery		9	297	154-486
Gastrectomy		4	164	124-257
Other		4	91	67-173
General surgery		35	96	82-117
Herniorrhaphy		18	97	79-127
Laparoscopic cholecystectomy		8	91	83-99
Orificial surgery		4	66	49-78
Gastroesophageal reflux surgical treatment		2	170	148-170
Other		3	106	74-106
Head and neck surgery		34	129	97-157
Thyroidectomy/ parathyroidectomy		17	125	113-148
Neck dissection		3	242	106-242
Partial parotidectomy with facial nerve conservation		3	153	150-153
Other		11	87	66-135
Gynecology and obstetrics (endometriosis)		23	118	77-197
Hematology (bone marrow biopsies)		12	37	30-41
Thoracic surgery		11	268	157-305
Oral and Maxillofacial Surgery		11	135	107-191
Jaw arthroplasty		4	109	77-179
Hemimandibulectomy or segmental resection		2	120	106-120
Jaw osteoplasty		2	114	107-114
Other		3	195	146-195
ERCP		10	82	71-90
Plastic surgery		3	186	160-186
Otorhinolaryngology		1	235	

Of the 320 cases analyzed, 109 were scheduled as the first procedures of day, and the remaining 211 cases at subsequent times (non-first time) (Table 3).

**Table 3:** Description of the data on delays of the total number of cases evaluated.

General OR room entrance (n=320)	General	1st scheduled	Other times
N	320	109	211
Delayed	246 (76,9%)	98 (89,9%)*	148 (70,1%)
On time	74 (23,1%)	11 (10,1%)	63 (29,9%)

*p<0,001

Considering the tolerance time of up to 15 minutes for the patient to enter the operating room according to the schedule on the map, we observed a delay rate of 89.9% in the first procedure. The average delay time was 39 min. For comparative purposes, the analysis of subsequent cases showed a lower delay rate, 70.1% (p<0.001), in relation to the time scheduled. 

Among patients set for the first procedure of the day, 57.8% (63) were on preoperative PAR more than 10 minutes in advance, and 42.2% (46), up to 10 minutes in advance, showing greater punctuality in the group present with more advance notice on PAR (p<0.001).

Table 4 brings the post-surgical hospital length of stay by specialty and surgical intervention subgroups within specialties

**Table 4:** Post-surgical hospital length of stay according to specialty and to types of surgical intervention within specialties.

Specialties and types of intervention	n	Postoperative dwell time (hours)	
		Median	IQR
Urology	83	17.4	7.6-24.2
Laparoscopic radical prostatectomy	20	24.4	21.0-28.7
Bladder tumor - endoscopic resection	15	13.8	7.2-20.3
Prostate electrovaporization	9	9.7	5.9-22.4
Specialties and types of intervention	n	Postoperative dwell time (hours)	
		Median	IQR
Percutaneous nephrolithotripsy	9	21.9	15.1-23.4
Nephrectomy	6	19.9	17.0-22.1
Unilateral flexible ureterorrenolithotripsy	6	6.2	31.-11.8
Cystoscopy and/or urethroscopy	5	4.7	4.1-9.0
Endoscopic resection of the prostate	4	24.3	10.4-54.1
Radical cystectomy	2	166.8	139.8-166.8
Other	7	7.6	3.4-12.0
Mastology	58	15.0	8.2 -18.9
Quadrantectomy with or without lymphadenectomy	35	12.2	7.0-17.6
Radical mastectomy	8	16.1	11.1-19.0
Mastectomy or quadrantectomy with immediate reconstruction	7	19.0	15.8-22.0
Excision of breast lesion	5	12.0	9.0-16.6
Other	3	16.2	4.9-16.2
Oncological surgery	39	60.9	20.4-96.3
Colectomy and rectosigmoidectomy	12	81.9	63.3-103.1
Oncogynecological surgery	10	21.9	19.9-24.6
Hepato-pancreatic surgery	9	68.2	32.2-129.2
Gastrectomy	4	118	39-162
Other	4	13.9	4.0-107.1
General surgery	35	8.4	5.7-15.2
Herniorrhaphy	18	7.1	5.0-13.0
Laparoscopic cholecystectomy	8	8.0	7.0-11.0
Orificial surgery	4	9.5	6.9-10.6
Gastroesophageal reflux surgical treatment	2	14.7	13.0-14.7
Other	3	38.2	12.5-38.2
Head and neck surgery	34	17.5	12.2-21.2
Thyroidectomy/parathyroidectomy	17	19.0	15.0-21.3
Neck dissection	3	39.4	17.4-39.4
Partial parotidectomy with facial nerve conservation	3	16.5	12.9-16.5
Other	11	9.3	5.6-20.2
Gynecology and obstetrics (endometriosis)	23	18.8	7.5-26.5
Hematology (bone marrow biopsies)	12	5.3	2.2-11.0
Thoracic surgery	11	69.5	22.0-75.4
Oral and Maxillofacial Surgery	11	12.8	7.4-18.2
Jaw arthroplasties	4	8.0	4.9-11.7
Hemimandibulectomy or segmental resection	2	10.9	3.7-10.9
Jaw osteoplasty	2	22.1	7.4-22.1
Other	3	16.4	13.4-16.4
ERCP	10	9.3	5.2-17.8
Plastic surgery	3	14.9	11.2-14.9
Otorhinolaryngology	1	47.3	47.3

Length of stay was measured in hours after post-anesthetic recovery. Radical cystectomy had the longest median postoperative stay (166h), followed by colectomy and rectosigmoidectomy (81.9h).

## DISCUSSION

The potential applicability of indoor location technology, combined with software to support steps during the patient’s trajectory, provides two tools for managers: one is an online panel that enables global communication for corrective or preventive activities in real-time, and the other offers global data for analysis and management of the hospital unit and its specific sectors.

Quick, assertive information is crucial for decision making. By tracking patient movement with beacons instead of relying on third-party sources, professional bias in recording activity times is eliminated. This automated data, based on the patient’s actual presence, provides reliable and objective information on hospital stays.

In recent years, technological advances related to automation and the use of artificial intelligence applications, combined with the use of mathematical models in applied research seeking to increase operational efficiency, have led to greater knowledge and understanding of process management, thus improving evidence-based decision-making^9^. 

In this regard, the fusion of internet-based technologies such as smart terminals, cloud computing, WI-FI, 5G, and interactive applications has paved the way for innovative solutions. Exploring and applying these technologies can help optimize health service processes, enable intelligent monitoring of service delivery quality, and provide increased understanding of health care aspects, which may inform future changes. Data gathering via digital innovation platforms has brought good results for monitoring the positioning of people and equipment in health facilities^13^. These studies reinforce our findings, in which the screening model demonstrated good effectiveness in the temporal location of patients in hospital sectors. The use algorithms that accurately incorporate procedure and transport times, room turnover, and hospital stay durations can optimize scheduling maps to make the best use of available resources during planned activity periods. It becomes possible to more accurately determine optimal opening hours, adjust staffing requirements for each period, and identify effective combinations of procedures to maximize installed capacity. This approach can help address bottlenecks that hinder adherence to surgical schedules and may reduce underutilization of operating rooms and the unit, thereby support increased patient admissions and improving the allocation of hospital resources.

As it is a hospital with a verticalized operator, the organization of specific processes and constant groups of medical professionals provides the study and delineation of hospital resources consumption patterns. As time passes and casuistry increases, the use of the indoor location tool participates in this knowledge’s refinement, bringing robustness in the determination of operating room use in minutes according to each procedure and hospital stay in hours for these patients. This refinement will allow greater efficiency in resource use, with more precise planning of the entry of elective patients in leaner flows, greater accuracy of arrival time, execution of the procedure, and occupation of hospital beds. 

Halim et al.^12^ conducted a literature review describing the importance of improving the punctuality of scheduled surgeries, reporting that in a study of Operating Rooms in the United Kingdom the adequate room usage was 73% of the planned time, with an average daily loss of two hours, which would be equivalent to 280,000 elective surgeries in one year, demonstrating, with tangible data, the impact on care and on hospitals financial loss. This efficiency indicator cannot be minimized, and the search for strategies has also been proposed in the literature due to increasing costs and decreasing profits associated with these surgical units^12^. 

Similarly, other authors describe how to start the day’s procedures on time, which is one of the targets sought in the efficient management of Operating Rooms. According to Lee et al.^3^, there is an average delay of 30 minutes in 50-80% of the first cases in tertiary hospitals. 

The execution on time of the first scheduled surgical procedure is one of the main efficiency indicators of Operating Rooms described in the literature. 

In our study, punctuality was defined as the first procedures starting up to 15 minutes after the scheduled time and without tolerance times for the other scheduled procedures in the day. We considered this tolerance sufficient to capture significant data, and the mean delay time was 39 minutes.

In this sense, evolutionary learning with the visibility of processes within each hospital unit makes performance indicators adjust constantly and continuously. This indoor location technology through BLE is a viable and less costly possibility than RFID technology, due to the ubiquity of bluetooth capture devices present on a large scale within hospital environments^13^. 

In addition, its installation and use requires training on a smaller scale of hospital employees and the information is automated. Many hospital situations may be incorporated according to the institution’s findings. For example, assessing the time of arrival of elective patients at the hospital may provide understanding of the ideal interval so that delays are not promoted, while at the same time not overloading the admission sector. 

This information is valuable for tertiary hospitals that share their beds with urgent and emergency sectors and work with a centralized bed management sector. This technology may allow the collection of information that can help bed management, for example, in data-based decision-making for prioritizing beds in hours, attending to emergency cases without harming the flow of elective surgeries, focusing on the balance of service efficiency and rational use of resources, with low idleness. In this sense, one should observe and pursue the effort to avoid underutilization or overutilization of the operating room by planning its optimization^5^ ^,^ ^13^ ^,^ ^14^.

As limitations of the study, we can report the dependence on the allocation of information by its executors at the correct time for the collection of reliable information. Another point of attention is the large number of participants in the various stages that require monitoring, making the training process more time-consuming, with the need for constant maintenance so that the information is standardized. Exemplified in our study, where sectors were smaller and with more closed teams (reception and preoperative preparation sector), the execution of activities in the application had higher compliance rates (75.6% and 72.5%, respectively), which did not happen in the subsequent stages of the other sectors. 

Another limitation was not tracking urgent and emergency surgeries, which meant that room occupancy data could not be used for analysis (nor room turnover), considering that during the day, elective and urgent patients rotate in the same room frequently. This technology, herein directed to the management of the operating room, was evolving and is progressing to concomitant mapping of non-elective patients.

In short, as it is the implementation of new technology within a hospital environment, there is a need to deepen training strategies and evaluate the execution of these steps in real time. However, even with the need for greater training, the collection of information on the times in each execution phase provides important data in the equalization of Service Level Agreements within the hospital teams. The combination of information with adequate training can make the surgical process more robust, efficient, and financially balanced, favoring the maintenance of quality health services. 

## CONCLUSION

Indoor location technology has applicability in the management of surgical patients in the hospital environment and brings useful data to support decision-making, facilitating the identification of bottlenecks, their causes, and enabling actions to improve processes.
